# An Approach to Improve Accuracy of Optical Tracking Systems in Cranial Radiation Therapy

**DOI:** 10.7759/cureus.239

**Published:** 2015-01-07

**Authors:** Patrick Stüber, Benjamin Wagner, Tobias Wissel, Ralf Bruder, Achim Schweikard, Floris Ernst

**Affiliations:** 1 University of Lübeck, Institute for Robotics and Cognitive Systems; 2 Institute for Robotics and Cognitive Systems, University of Lübeck; 3 Institute for Robotics and Cognitive Systems, University of Lubeck,, Institute for Robotics and Cognitive Systems, University of Lubeck,; 4 Institute for Robotics and Cognitive Systems, University of Luebeck, Institute for Robotics and Cognitive Systems, University of Lubeck

**Keywords:** optical head tracking, estimation of soft tissue thickness

## Abstract

This work presents a new method for the accurate estimation of soft tissue thickness based on near infrared (NIR) laser measurements. By using this estimation, our goal is to develop an improved non-invasive marker-less optical tracking system for cranial radiation therapy. Results are presented for three subjects and reveal an RMS error of less than 0.34 mm.

## Introduction

The combination of high acquisition rates with high accuracy has made optical tracking systems more and more popular for a wide range of medical applications. Especially in radiation therapy, tracking systems provide a high potential to replace conventional immobilization techniques and x-ray based localization systems to minimize the radiation dose. Although many commercial systems for surface recognition are available, a target localization accuracy in sub-millimeter range has not been achieved yet. The optical properties of soft tissue especially affect the accuracy.

Our research focuses on the development of a non-invasive markerless optical tracking system for cranial radiation therapy. For this purpose, we want to combine 3D surface information using a commonly used triangulation technique with tissue thickness information from a novel optical technique. Here, we present first 4D tomograms from three human foreheads (Video 1).

**Video 1:** An approach to improve accuracy of optical tracking systems in cranial radiation therapy

## Materials and methods

Initial Monte Carlo simulations showed that near infrared (NIR) light delivers the best trade-off between absorption and scattering to maximize the penetration depth [1]. At a wavelength of *λ* = 850nm, it is especially possible to achieve a penetration depth of up to 20 mm. Furthermore, these simulations show that the backscattered light structure of a projected laser spot is characteristic for each tissue thickness. Therefore, it is possible to estimate the tissue thickness by analyzing the backscattered signal.

Experimental setup and acquisition of a tomogram

**Figure 1:**
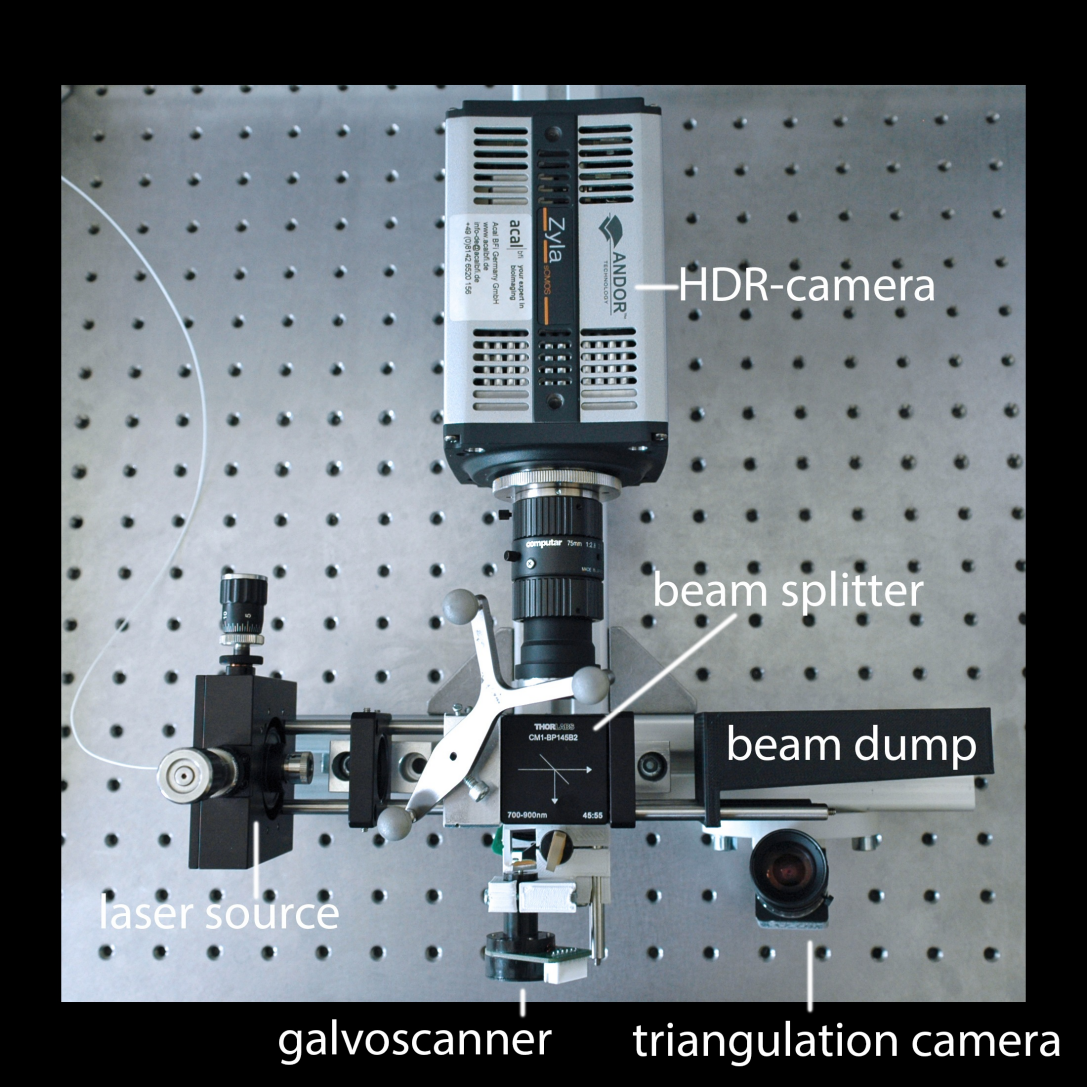
NIR scanner setup containing a laser source, a beam splitter, a beam dump, a galvanometric scanning unit, a triangulation camera, and a HDR camera

To estimate the tissue thickness from near infrared (NIR) scans a special measurement setup was designed (Figure 1). Wissel, et al. and Stüber, et al. published a detailed description [2-3]. The setup contains a laser source (Thorlabs LPS-830-FC) (Thorlabs, Inc, Newton, NJ), a galvanometric scanner system (Galvo-System AXJ-V20, hereafter: galvoscanner), and two cameras. One camera has a high dynamic range contrast (Andor Zyla, hereafter: *HDR-camera*) (Andor Ltd, Belfast, N. Ireland) and the other camera (IDS µEye UI-3240CP) (IDS GmbH, Obersulm, Germany) is used for triangulation.

Light coming from the laser source is deflected by the galvoscanner which pointwisely projects a 32 x 32 grid on the forehead of a subject. The laser spot has a diameter of 1.3 mm. The distance between adjacent columns is approx. 1.5 mm and between adjacent rows approx. 1 mm. The HDR-camera is aligned such that the optical axis of the camera overlays the laser beam axis. We call this alignment *“inBeam”*-perspective. The HDR- and the triangulation camera collect backscattered light from the forehead.

From the captured images of the HDR-camera, optical features are determined from concentric regions of interest (ROI) around the laser spot. These features correlate with the tissue thickness (1D information) as described by Wissel, et al. in 2013 [1]. The second camera is used to triangulate each laser point (3D information). For triangulation, the centroids of the imaged laser points are detected. A set of calibration data, which describes the geometric relations between the camera and the galvoscanner, was acquired in advance. By using the calibration data and the detected centroids, the corresponding spatial points are reconstructed by triangulation. Wagner, et al. [4] published the entire procedure for highly accurate reconstruction of surfaces. By combining both datasets, it is possible to determine a 4D tomogram of the forehead.

MRI scans

To convert the acquired optical features into an estimation of the tissue thickness, a volumetric scan of the subject’s forehead is required as ground truth. For this purpose, we performed T1-weighted MRI scans with a Philips Achieva 3.0T scanner (Philips Eindhoven, The Netherlands), whereby the in-plane resolution was 0.15 mm and the out-of-plane resolution was 1 mm. The MRI-volume was aligned to the anterior commissure - posterior commissure line (AC-PC line). Because the focus of these scans lies on the tissue thickness extraction, we developed a special procedure to simplify the semi-automatic segmentation process. Further details have been published by Wissel, et al. [2]. Since the matching errors for all three subjects are less than 0.65 mm, we assume that distortion effects during the MRI acquisition are negligible.

Matching of MRI and NIR scans

**Figure 2:**
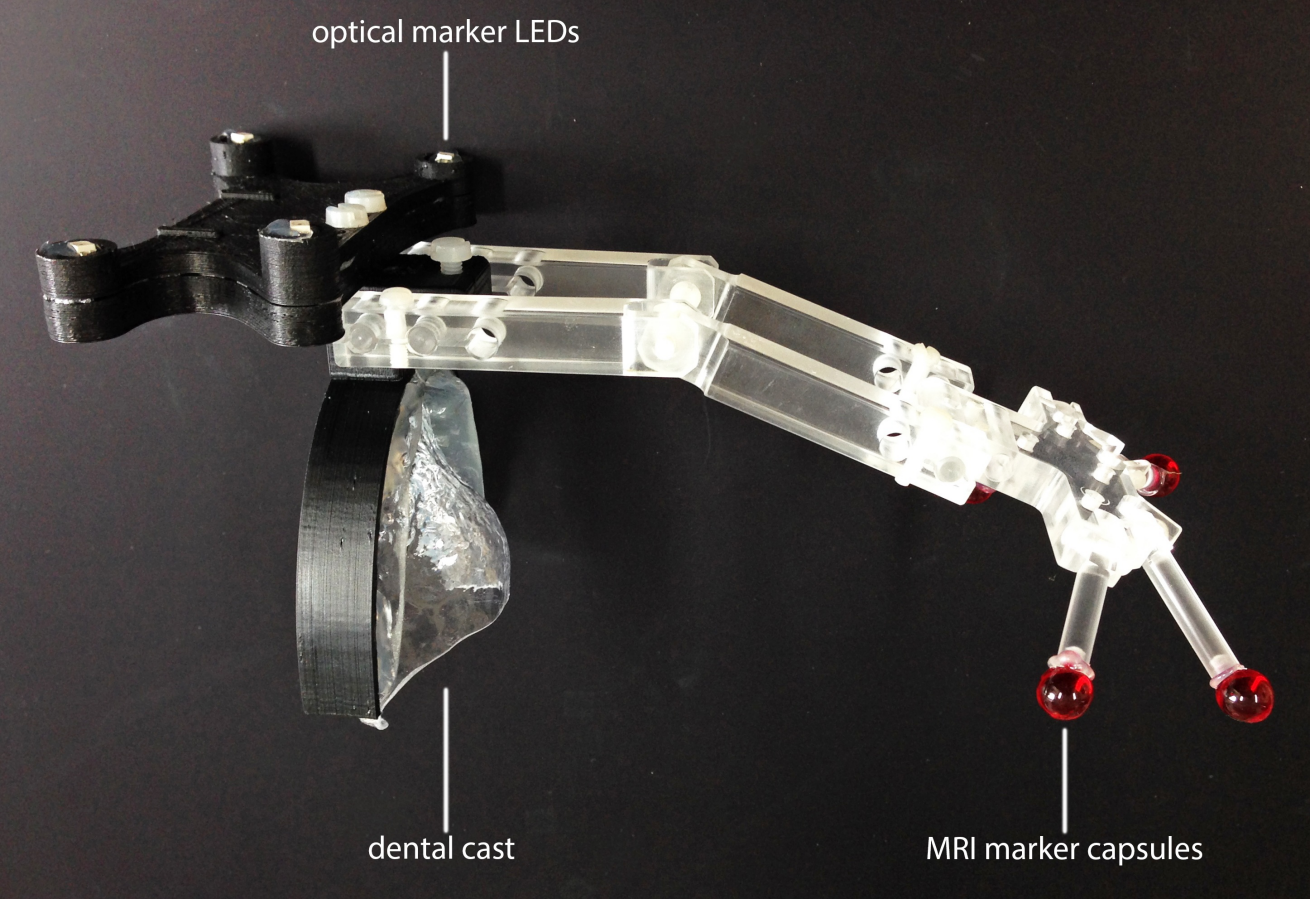
Marker geometry utilized for the matching of MRI and NIR scans

To guarantee the most reliable matching of MRI data and NIR scans, we used a customized marker (Figure 2) which can be detected in the MRI scans. Furthermore, this marker can also be detected by the accuTrack 250 optical tracking system (atracsys, Le Mont-sur-Lausanne, Switzerland). A dental cast fixed the marker in a reproducible position in relation to the subject’s forehead. An additional CT scan of the marker geometry was acquired, and the marker spheres were extracted from the CT and MRI scan. This is necessary to establish a relation between MRI and NIR scans. By tracking the marker during the optical measurement, the pre-alignment between the NIR and the MRI scan was achieved. Afterwards, the alignment between both scans was refined by using the Iterative Closest Point (ICP) algorithm [5].

Machine learning process

To convert the acquired optical features into an estimation of the tissue thickness, a supervised machine learning model (Gaussian Process model (GP)) [6] using a *k_ARD_*-kernel) was trained on the MRI data and the NIR data. Thereby, the amount of NIR data points given in Table 1 were split up into ten equal subsets. The GPs were then trained on one subset and applied to the remaining nine subsets to estimate the thickness of the soft tissue. Afterwards, the GPs were trained again on the next subset and applied to the rest. This cross validation was repeated five times. For each tissue thickness estimation, the errors were determined. Finally, the training process involved five times tenfold cross validation.

**Table 1:** Accuracy of matching and soft tissue thickness estimation. The values represent the number of valid points and the RMS in mm.

	Subject 1 (S1)	Subject 2 (S2)	Subject 3 (S3)
Data samples	888	945	790
ICP	0.65	0.47	0.50
GP: k_ard, with angle_	0.33	0.33	0.28

Note that the incident angle was considered as an additional feature during the machine learning process to comprise the angular influence.

## Results

The described method was carried out for three subjects. Informed patient consent was obtained for our measurements. Using the marker geometry in Figure 2, the acquired NIR scans were pre-aligned to the MRI scans. An ICP refinement was then carried out. Table 1 shows the entire root-mean-square (RMS) errors after both alignment steps. A low matching error of less than 0.65 mm was achieved for all three subjects.

**Figure 3:**
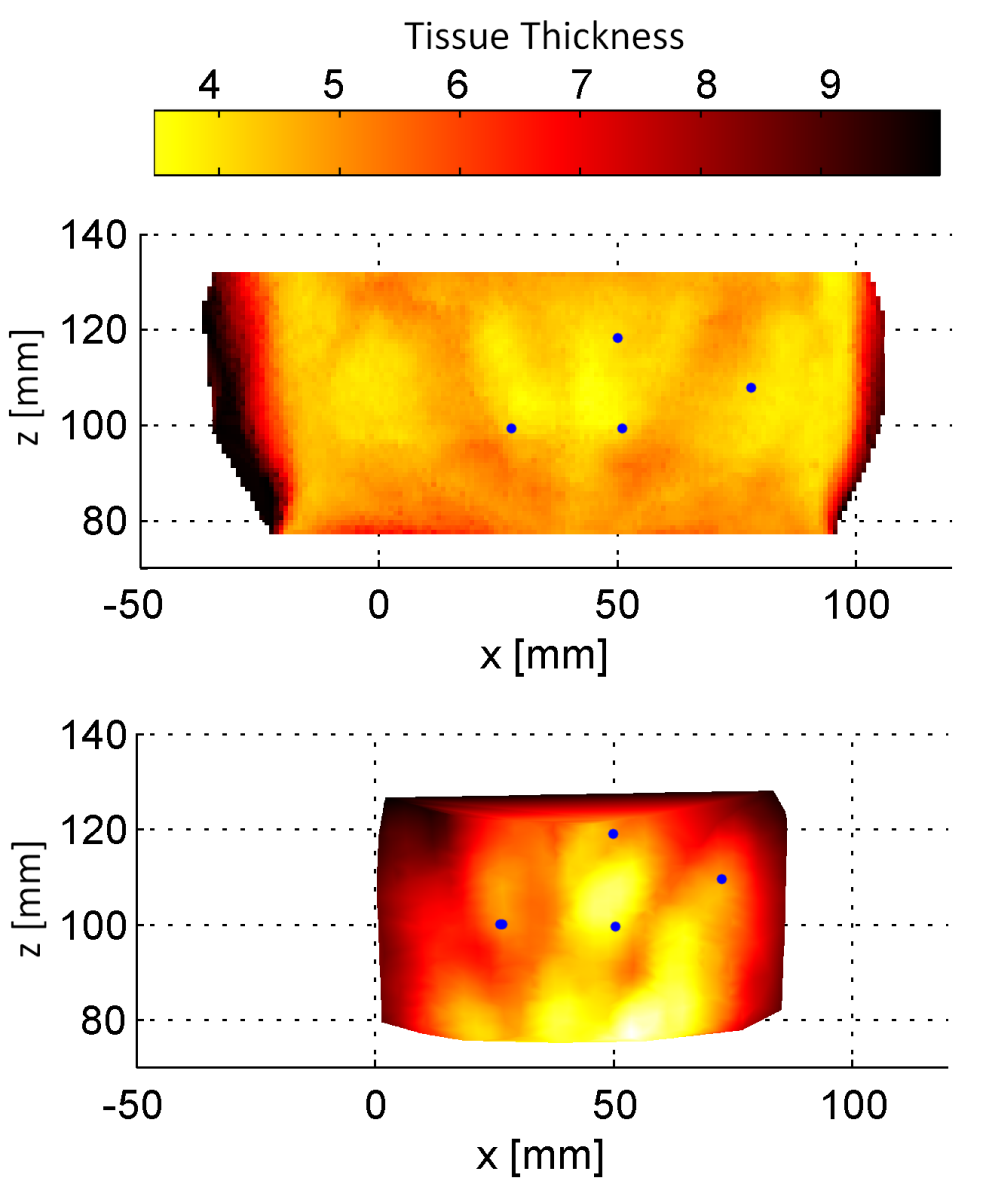
2D projection of the matching between the MRI scan (top) and NIR scan (bottom) for Subject 1. The MRI scan contains the thickness of the soft tissue, and the NIR scan contains one of the acquired NIR features. The blue landmarks are provided for orientation.

The matching result for Subject 1 is presented in Figure 3. It is important to consider that the color code for the MRI represents the extracted tissue thickness, whereas the color code for the NIR scan represents the reflectivity. Both are negatively correlated as shown by Wissel, et al. [7]. Subcutaneous structures are partially visible in both scans and marked by artificial landmarks (Figure 3). The influence of the incident angle of the laser beam can be seen in the outer regions of the NIR scan. The angular influence leads to darker regions in the NIR scan as published by Stüber, et al. [8].

The results of the machine learning-based model for tissue thickness estimation are given in Table 1 and are represented by the RMS error. A very low estimation error of less than 0.34 mm was achieved for all three subjects.

## Discussion

The results show a low matching error for all three subjects, which is the basis for further processing of MRI and NIR data. A low matching error increases the correlation of MRI and NIR data and consequently leads to very accurate results for the estimation of the soft tissue thickness.

The matching error would increase with a change of the surface (e.g. wrinkles and facial expressions). To avoid such effects, the NIR scans were performed directly after the MRI scans. The subjects were instructed to avoid facial expressions.

The results define the basis for a new optical head tracking method that is capable of compensating for the soft tissue. Thus, we expect that this new method will lead to very accurate tracking results.

## Conclusions

This paper describes a new method for the accurate estimation of soft tissue thickness based on NIR measurements. Results are presented for three subjects and show an RMS error of less than 0.34 mm. These promising results are the basis for the development of a new highly accurate optical head tracking method.
